# Ghrelin Represses Thymic Stromal Lymphopoietin Gene Expression through Activation of Glucocorticoid Receptor and Protein Kinase C Delta in Inflamed Skin Keratinocytes

**DOI:** 10.3390/ijms23073977

**Published:** 2022-04-02

**Authors:** Hayan Jeong, Hyo-Jin Chong, Jangho So, Yejin Jo, Tae-Young Yune, Bong-Gun Ju

**Affiliations:** 1 Department of Life Science, Sogang University, Seoul 04107, Korea; hayan90@sogang.ac.kr (H.J.); chd9388@sogang.ac.kr (H.-J.C.); hojangso@naver.com (J.S.); basegirl0706@icloud.com (Y.J.); 2 Age-Related and Brain Diseases Research Center, Kyung Hee University, Seoul 02447, Korea; tyune@khu.ac.kr; 3 Department of Biochemistry and Molecular Biology, School of Medicine, Kyung Hee University, Seoul 02447, Korea

**Keywords:** atopic dermatitis, thymic stromal lymphopoietin, ghrelin, glucocorticoid receptor, NF-κB, p300, PKCδ

## Abstract

Ghrelin, a peptide hormone secreted from enteroendocrine cells of the gastrointestinal tract, has anti-inflammatory activity in skin diseases, including dermatitis and psoriasis. However, the molecular mechanism underlying the beneficial effect of ghrelin on skin inflammation is not clear. In this study, we found that ghrelin alleviates atopic dermatitis (AD)-phenotypes through suppression of thymic stromal lymphopoietin (TSLP) gene activation. Knockdown or antagonist treatment of growth hormone secretagogue receptor 1a (GHSR1a), the receptor for ghrelin, suppressed ghrelin-induced alleviation of AD-like phenotypes and suppression of TSLP gene activation. We further found that ghrelin induces activation of the glucocorticoid receptor (GR), leading to the binding of GR with histone deacetylase 3 (HDAC3) and nuclear receptor corepressor (NCoR) NCoR corepressor to negative glucocorticoid response element (nGRE) on the TSLP gene promoter. In addition, ghrelin-induced protein kinase C δ (PKCδ)-mediated phosphorylation of p300 at serine 89 (S89), which decreased the acetylation and DNA binding activity of nuclear factor- κB (NF-κB) p65 to the TSLP gene promoter. Knockdown of PKCδ abolished ghrelin-induced suppression of TSLP gene activation. Our study suggests that ghrelin may help to reduce skin inflammation through GR and PKCδ-p300-NF-κB-mediated suppression of TSLP gene activation.

## 1. Introduction

Atopic dermatitis (AD) is a chronic skin inflammatory disease that is characterized by xerosis, intense pruritus, and eczema [1,2]. The estimated prevalence of AD in the worldwide population is 10~20% of children and 2~10% of adults [3,4]. AD disrupts a patient’s quality of life through sleep disturbance, and AD is associated with other atopic disorders such as food allergy, allergic rhinitis, and asthma [5,6]. Although topical medications, including corticosteroids and calcineurin inhibitors, are widely used, they have undesirable side effects, which suggest the need for medications with higher efficacy and safety [7,8,9,10]. Although the etiology of AD is very complicated, currently, it is believed that epidermal barrier abnormalities and immune dysfunctions are the main reasons for the pathogenesis [11,12,13].

Thymic stromal lymphopoietin (TSLP), an IL-7-like cytokine, plays a key role in the development and progression of allergic diseases [14,15]. TSLP is highly expressed in lesional skin of AD patients and induces severe itching by directly activating TRPA1-positive sensory neurons [16,17,18]. Recently, anti-TSLP monoclonal antibody has been developed for inflammatory skin diseases and asthma [19,20]. Given the importance of TSLP in allergic diseases, the molecular mechanism of TSLP gene regulation has been studied. For example, ablation of the nuclear receptors retinoid X receptor (RXR)-α and RXR-β or topical application of vitamin D3 on mouse skin induces TSLP expression [21,22]. In addition, multiple transcription factors, including NF-κB, activator protein 1 (AP-1), signal transducer and activator of transcription (STAT), and mothers against decapentaplegic homology (SMAD), have been identified for the control of TSLP expression [23,24,25,26].

Ghrelin is an orexigenic peptide hormone, which is mainly produced in the stomach during fasting and then released into systemic circulations [27,28]. Ghrelin participates in various biological processes, including food intake, energy homeostasis, and tissue regeneration through interacting with and stimulating the growth hormone secretagogue receptor type 1a (GHSR1a) [29,30,31,32]. In addition, ghrelin has been found to suppress the production of pro-inflammatory cytokines by inhibiting the NF-κB activity in immune and endothelial cells [33,34,35]. Similarly, ghrelin has a therapeutic effect on contact dermatitis and psoriasis by antagonizing the NF-κB pathway [34,36]. Thus, ghrelin has attracted attention as a novel therapeutic intervention in diverse inflammatory diseases [37,38,39]. However, the exact mechanisms underlying the anti-inflammatory activity of ghrelin in inflammatory skin diseases such as AD remain largely unknown.

## 2. Results

### 2.1. Ghrelin Alleviates AD-like Phenotypes and Suppresses TSLP Gene Activation

**Figure 1:**
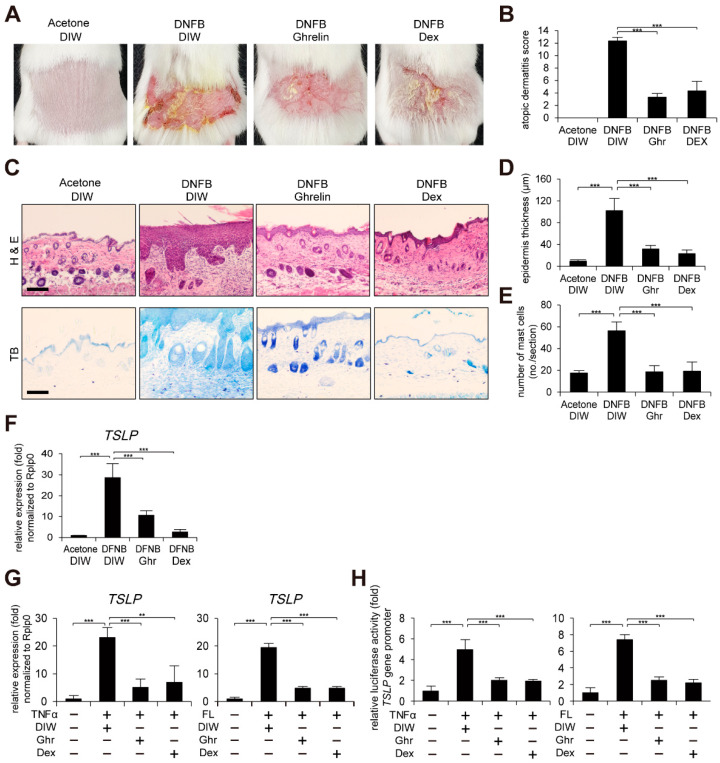
Ghrelin alleviates AD-like phenotypes and suppresses TSLP gene activation. (**A**) Topical application of ghrelin alleviates AD-like phenotypes induced by DNFB treatment (see Section 4) (*n* = 6/group). Dexamethasone (Dex) was used as positive control. Acetone and deionized water (DIW) were used as solvent for DNFB and ghrelin, respectively. Representative images are shown. (**B**) Ghrelin reduces the severity of AD-like phenotypes. The severity was determined using a scoring index of AD (see Section 4). (**C**) Skin tissue sections were stained with hematoxylin and eosin (H&E) or toluidine blue (TB). Scale bar, 100 µm. Representative images are shown. (**D**) Ghrelin decreases epidermis thickness in AD-like skin lesions. Epidermis thickness was measured in H&E stained tissue sections (*n* = 6/group). (**E**) Ghrelin decreases infiltration of mast cells in AD-like skin lesions. The number of mast cells was counted from 5 randomly selected low-power fields in TB stained tissue sections (*n* = 6/group). (**F**) Ghrelin suppresses TSLP gene activation in AD-like skin lesions. Real-time PCR was performed to quantify TSLP and ribosomal protein lateral stalk subunit P0 (RPLP0) transcripts from skin (*n* = 6/group). RPLP0 was used as a control. (**G**) Ghrelin suppresses TSLP gene activation in HaCaT keratinocytes treated with TNFα or bacterial flagellin (FL). DIW was used as solvent for TNFα, flagellin, and ghrelin. Real-time PCR was performed to quantify TSLP and RPLP0 transcripts (*n* = 3). (**H**) Ghrelin suppresses TSLP gene promoter activity in HaCaT keratinocytes treated with TNFα or bacterial flagellin. After HaCaT keratinocytes were transiently transfected with luciferase reporter vector containing the TSLP gene promoter and control Renilla luciferase expression vector, luciferase activity was measured (*n* = 3). All data represent mean ± S.E.M. Significance values were ** *p* ≤ 0.01 and *** *p* ≤ 0.005.

Given the anti-inflammatory effect of ghrelin in diverse diseases such as brain, skin injury, sepsis, and gastritis [34,38,39,40,41], we investigated whether ghrelin alleviates AD-like phenotypes using a mouse model induced by 1-fluoro-2,4-dinitrobenzene (DNFB) treatment (see Section 4). Dexamethasone was topically applied as a positive control. Using an AD scoring index, we measured the skin severity as a sum of the following four symptoms: hemorrhage, swelling, erosion, and dryness (See Section 4). Topical application of ghrelin alleviated DNFB-induced dermatitis (Figure 1A,B). Histological examinations showed that ghrelin suppresses DNFB-induced skin hyperplasia (Figure 1C,D). We also found decreased infiltration of mast cells by ghrelin treatment (Figure 1C,E). Ghrelin further suppressed TSLP, IL-4, IL-10, IL-13, IL-22, IL-25, IL-31, IL-33, and colony stimulating factor 2 (CSF2) gene activation in AD-like skin lesions (Figure 1F and Figure S1). Using a cell model, we consistently found that ghrelin suppresses TSLP gene activation and the promoter activity in HaCaT keratinocytes treated with tumor necrosis factor α (TNFα) or bacterial flagellin (Figure 1G,H).

### 2.2. GHSR1a Is Required for Ghrelin-Induced Suppression of TSLP Gene Activation

**Figure 2:**
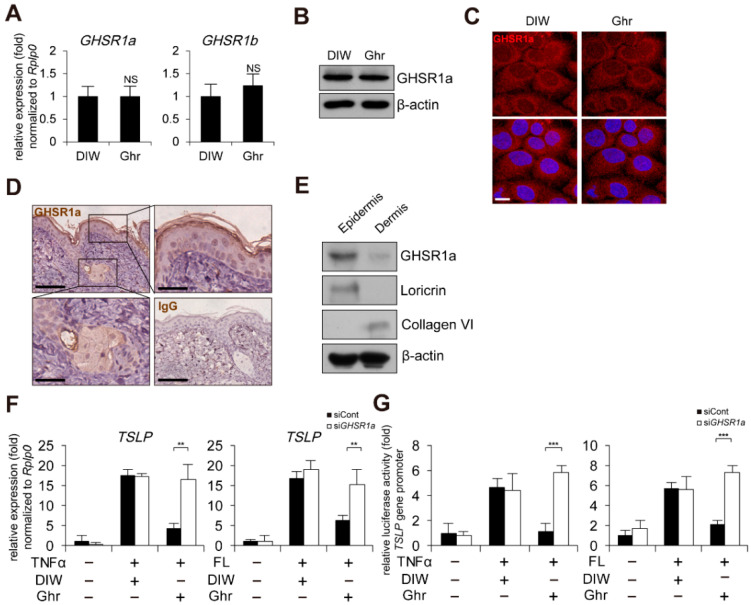
GHSR1a is required for ghrelin-induced suppression of TSLP gene activation. (**A**) Ghrelin does not alter GHSR1 expression in HaCaT keratinocytes. Real-time PCR was performed to quantify GHSR1a, GHSR1b, and RPLP0 transcripts (*n* = 3). RPLP0 was used as a control. DIW was used as solvent for ghrelin. NS, not significant. (**B**) HaCaT keratinocyte lysates were immunoblotted with anti-GHSR1a and anti-β-actin antibodies (*n* = 3). β-actin was used as a control. Representative images are shown. (**C**) HaCaT keratinocytes were immunostained with anti-GHSR1a antibody (top panel). DAPI was used for nuclei staining (low panel). Scale bar, 10 μm. Representative images are shown. (**D**) GHSR1a is expressed in epidermis and hair follicles of mouse skin. Epidermis and hair follicles were magnified (scale bar, 25 μm). Skin tissue sections were immunostained with anti-GHSR1a antibody. IgG was used as a negative control. Scale bar, 50 μm. Representative images are shown. (**E**) GHSR1a is expressed in mouse skin epidermis. Epidermis and dermis were isolated from skin. Skin lysates were immunoblotted with anti-GHSR1a, anti-loricrin, anti-collagen VI, and anti-β-actin antibodies (*n* = 3). Loricrin and collagen VI were used as specific markers for epidermis and dermis, respectively. β-actin was used as a control. Representative images are shown. (**F**) Depletion of GSHR1a by siRNA abolishes ghrelin-induced suppression of TSLP gene activation in HaCaT keratinocytes treated with TNFα or bacterial flagellin (FL). After keratinocytes were transfected with control (siCont) or GHSR1a siRNA (siGHSR1a), real-time PCR was performed to quantify TSLP and RPLP0 transcripts (*n* = 3). RPLP0 was used as a control. DIW was used as solvent for TNFα, flagellin, and ghrelin. (**G**) Depletion of GSHR1a by siRNA abolishes ghrelin-induced suppression of TSLP gene promoter activity in HaCaT keratinocytes treated with TNFα or bacterial flagellin. After HaCaT keratinocytes were transiently transfected with luciferase reporter vector containing the TSLP gene promoter and control Renilla luciferase expression vector, luciferase activity was measured (*n* = 3). All data represent mean ± S.E.M. Significance values were ** *p* ≤ 0.01 and *** *p* ≤ 0.005.

To determine whether the anti-inflammatory effect of ghrelin is mediated through its receptor, growth hormone secretagogue receptor (GHSR) [27,42,43,44], we first examined the gene expression of a functional receptor, GHSR1a [45,46,47,48]. Ghrelin did not alter the expression of GHSR1a in mRNA and protein level in HaCaT keratinocytes (Figure 2A,B). GHSR1a was expressed in the cell membrane of HaCaT keratinocytes (Figure 2C). In mouse skin, we found positive signals for GHSR1a in epidermis and hair follicles (Figure 2D). In addition, GHSR1a was expressed in DNFB-induced AD-like mouse skin (Supplementary Figure S2). To clarify the epidermal expression of GHSR1a, epidermis and dermis were isolated from mouse skin, and Western blot analysis was performed. Consistent with the results from immunohistochemistry, GHSR1a was expressed in the epidermis, which is positive for loricrin expression (Figure 2E). We further confirmed the presence of GHSR1a by examination of ghrelin-induced calcium mobilization in HaCaT keratinocytes [49,50]. Although ghrelin-induced calcium influx, depletion of GHSR1a by siRNA reduced ghrelin-induced calcium influx (Supplementary Figures S3 and S4A). Next, we examined the effect of GHSR1a depletion on TSLP gene expression. As expected, depletion of GHSR1a by siRNA abolished ghrelin-induced suppression of TSLP gene activation and the gene promoter activity in HaCaT keratinocytes treated with TNFα or bacterial flagellin (Figure 2F,G).

### 2.3. GHSR1a Antagonist Reduces Ghrelin-Induced Alleviation of AD-like Phenotypes

**Figure 3:**
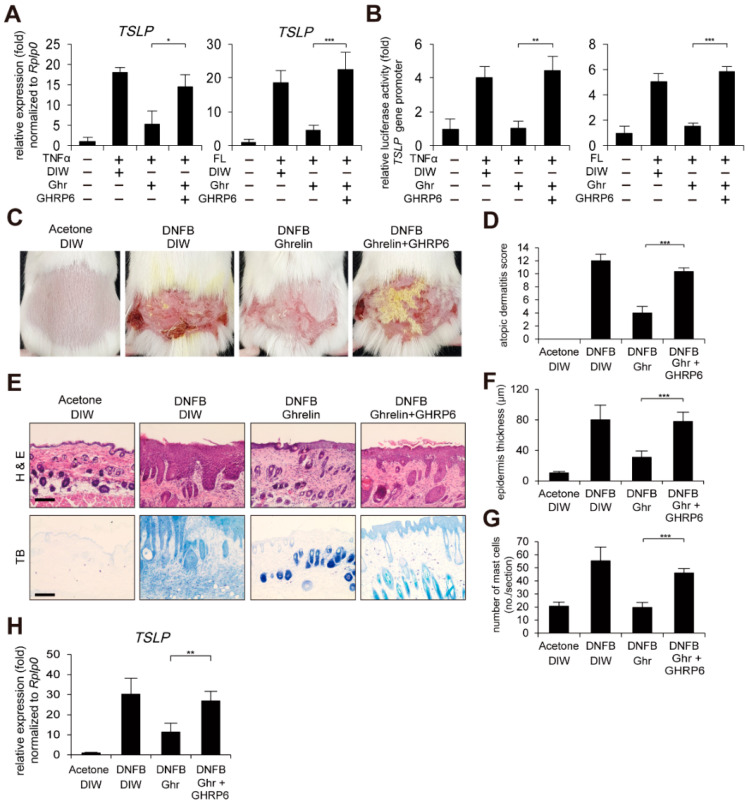
GHSR1a antagonist abolishes ghrelin-induced alleviation of AD-like phenotypes. (**A**) [D-Lys3]-GHRP6 (GHRP6), a GHSR1 antagonist, abolishes ghrelin-induced suppression of TSLP gene activation in HaCaT keratinocytes treated with TNFα or bacterial flagellin (FL). Real-time PCR was performed to quantify TSLP and RPLP0 transcripts (*n* = 3). RPLP0 was used as a control. DIW was used as solvent for TNFα, flagellin, ghrelin, and GHRP6. (**B**) GHRP6 abolishes ghrelin-induced suppression of TSLP gene promoter activity in HaCaT keratinocytes treated with TNFα or bacterial flagellin. After HaCaT keratinocytes were transiently transfected with luciferase reporter vector containing the TSLP gene promoter and control Renilla luciferase expression vector, luciferase activity was measured (*n* = 3). (**C**) GHRP6 abolishes ghrelin-induced alleviation of AD-like phenotypes. After mice were sensitized with DNFB for 7 days, DNFB was further topically applied to the shaved dorsal skin in combination with ghrelin or GHRP6 for 12 days (*n* = 6/group). Acetone was used as solvent for DNFB. DIW was used as solvent for ghrelin and GHRP6. Representative images are shown. (**D**) GHRP6 abolishes ghrelin-induced decrease in the severity of AD-like phenotypes. A scoring index of AD was used to determine the severity. (**E**) Skin tissue sections were stained with hematoxylin and eosin (H&E) or toluidine blue (TB). Representative images are shown. Scale bar, 100 µm. (**F**) GHRP6 abolishes ghrelin-induced decrease of epidermis thickness in AD-like skin lesions. Epidermis thickness was measured in H&E stained tissue sections (*n* = 6/group). (**G**) GHRP6 abolishes ghrelin-induced decrease in infiltration of mast cells in AD-like skin lesions. The number of mast cells was counted from 5 randomly selected low-power fields in TB stained tissue sections (*n* = 6/group). (**H**) GHRP6 abolishes ghrelin-induced suppression of TSLP gene activation in AD-like skin lesions. Real-time PCR was performed to quantify TSLP and RPLP0 transcripts from skin (*n* = 6/group). All data represent mean ± S.E.M. Significance values were * *p* ≤ 0.05, ** *p* ≤ 0.01, and *** *p* ≤ 0.005.

We next examined whether inhibition of GHSR1a has negative effects on ghrelin-induced alleviation of AD-like phenotypes using a GHSR1 antagonist, [D-Lys3]-growth hormone releasing peptide 6 (GHRP6) [36,51,52]. Consistent with the depletion of GHSR1a, GHRP6 suppressed ghrelin-induced suppression of TSLP gene activation and the gene promoter activity in HaCaT keratinocytes treated with TNFα or bacterial flagellin (Figure 3A,B). Topical application of GHRP6 abolished ghrelin-induced alleviated AD-like phenotypes such as AD score, skin hyperplasia, and infiltrated mast cells (Figure 3C–G). In addition, GHRP6 abolished the ghrelin-induced suppression of TSLP, IL-4, IL-10, IL-13, IL-22, IL-25, IL-31, IL-33, and CSF2 gene activation in DNFB-treated mouse skin (Figure 3H and Figure S5).

### 2.4. Ghrelin Suppresses TSLP Gene Activation through GR Activation

**Figure 4:**
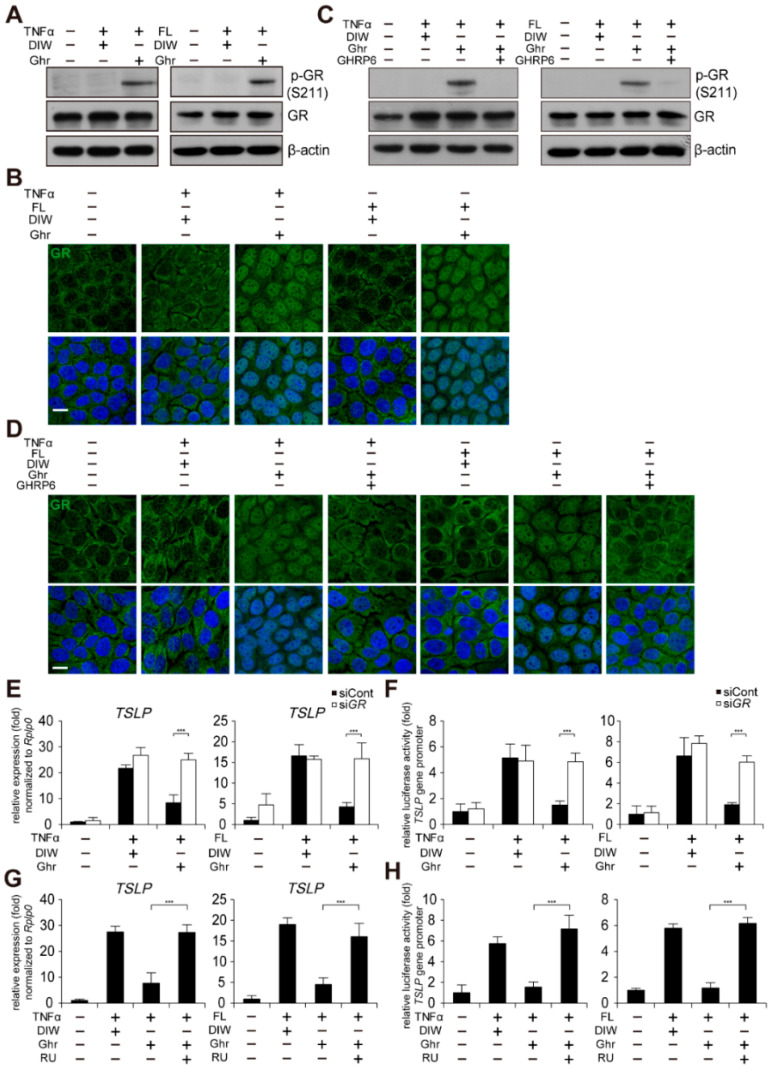
Ghrelin suppresses TSLP gene activation through GR activation. (**A**) Ghrelin induces phosphorylation of GR (S211) in HaCaT keratinocytes treated with TNFα or bacterial flagellin (FL). HaCaT keratinocyte lysates were immunoblotted with anti-GR, anti-phospho GR (S211), and anti-β-actin antibodies (*n* = 3). β-actin was used as a control. DIW was used as solvent for TNFα, flagellin, ghrelin, and GHRP6. Representative images are shown. (**B**) Ghrelin induces nuclear translocation of GR in HaCaT keratinocytes treated with TNFα or bacterial flagellin. HaCaT keratinocytes were immunostained with anti-GR antibody (*n* = 3) (top panel). DAPI was used for nuclei staining (low panel). Scale bar, 10 µm. Representative images are shown. (**C**) GHRP6 abolishes ghrelin-induced phosphorylation of GR (S211) in HaCaT keratinocytes treated with TNFα or bacterial flagellin. HaCaT keratinocyte lysates were immunoblotted with anti-GR, anti-phospho GR (S211), or anti-β-actin antibodies (*n* = 3). Representative images are shown. (**D**) GHRP6 abolishes ghrelin-induced nuclear translocation of GR in HaCaT keratinocytes treated with TNFα or bacterial flagellin. HaCaT keratinocytes were immunostained with anti-GR antibody (*n* = 3) (top panel). DAPI was used for nuclei staining (low panel). Scale bar, 10 µm. Representative images are shown. (**E**,**F**) Depletion of GR by siRNA abolishes ghrelin-induced suppression of TSLP gene activation in HaCaT keratinocytes treated with TNFα or bacterial flagellin. Real-time PCR was performed to quantify TSLP and RPLP0 transcripts (*n* = 3). RPLP0 was used as a control. After HaCaT keratinocytes were transiently transfected with luciferase reporter vector containing the TSLP gene promoter and control Renilla luciferase expression vector, luciferase activity was measured (*n* = 3). (**G**,**H**) RU486 (RU), a GR antagonist, abolishes ghrelin-induced suppression of TSLP gene activation in HaCaT keratinocytes treated with TNFα or bacterial flagellin. Real-time PCR was performed to quantify TSLP and RPLP0 transcripts (*n* = 3). After HaCaT keratinocytes were transiently transfected with luciferase reporter vector containing the TSLP gene promoter and control Renilla luciferase expression vector, luciferase activity was measured (*n* = 3). All data represent mean ± S.E.M. Significance value was *** *p* ≤ 0.005.

It has been previously reported that subcutaneous infusion of ghrelin increases GHSR1a-dependent glucocorticoid receptor (GR) expression in peritoneal macrophages from injured rat skin [34]. Thus, we examined the effect of ghrelin on GR activation in HaCaT keratinocytes treated with TNFα or bacterial flagellin. Although we did not observe elevation of GR expression by ghrelin (Figure 4A), ghrelin-induced phosphorylation of GR at serine 211 (S211) and nuclear localization of GR (Figure 4A,B). GHRP6 abolished ghrelin-induced phosphorylation of GR (S211) and nuclear localization of GR (Figure 4C,D). We next tested GR-dependent suppression of TSLP gene activation using siRNA against GR (Supplementary Figure S4B). Depletion of GR abolished ghrelin-induced suppression of TSLP gene activation and the gene promoter activity in HaCaT keratinocytes treated with TNFα or bacterial flagellin (Figure 4E,F). Consistently, RU-486 (also known as mifepristone), a GR antagonist, abolished ghrelin-induced suppression of TSLP gene activation and the gene promoter activity in HaCaT keratinocytes treated with TNFα or bacterial flagellin (Figure 4G,H).

### 2.5. Ghrelin Induces Recruitment of GR to nGRE Site on TSLP Gene Promoter 

**Figure 5:**
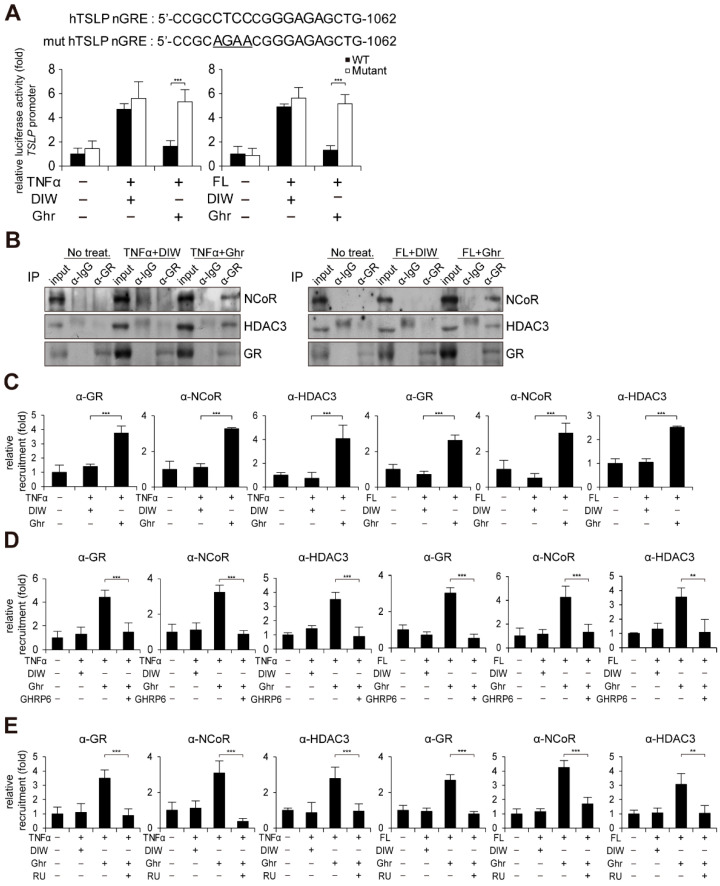
Ghrelin induces recruitment of GR to nGRE site on TSLP gene promoter. (**A**) Mutation of nGRE abolishes ghrelin-induced suppression of TSLP gene promoter activity in HaCaT keratinocytes treated with TNFα or bacterial flagellin (FL). After HaCaT keratinocytes were transiently transfected with luciferase reporter vector containing the TSLP gene promoter and control Renilla luciferase expression vector, luciferase activity was measured (*n* = 3). Mutated nucleotides are underlined. DIW was used as solvent for TNFα, flagellin, and ghrelin. (**B**) Ghrelin induces interaction of GR with NCoR and HDAC3 in HaCaT keratinocytes treated with TNFα or bacterial flagellin. HaCaT keratinocyte lysates were immunoprecipitated with the anti-GR antibody, then immunoblotted with anti-NCoR, anti-HDAC3, and anti-GR antibodies (*n* = 3). IgG was used as a control. Representative images are shown. (**C**) Ghrelin induces the recruitment of GR, NCoR, and HDAC3 to the nGRE site on the TSLP gene promoter. ChIP assay was performed using anti-GR, anti-NCoR, and anti-HDAC3 antibodies (*n* = 3). The occupancy of each protein was quantified using real-time PCR at the gene promoter encompassing the nGRE site. IgG was used for negative control. (**D**) GHRP6 abolishes ghrelin-induced recruitment of GR, NCoR and HDAC3 to the nGRE site on the TSLP gene promoter (*n* = 3). (**E**) RU486 (RU) abolishes ghrelin-induced recruitment of GR, NCoR, and HDAC3 to the nGRE site on the TSLP gene promoter (*n* = 3). All data represent mean ± S.E.M. Significance values were ** *p* ≤ 0.01 and *** *p* ≤ 0.005.

Previous reports and in silico analysis indicated the presence of negative glucocorticoid response element (nGRE) on the TSLP gene promoter [23,53,54,55]. Promoter reporter assay implied that ghrelin might suppress TSLP gene activation by binding GR to nGRE site on the TSLP gene promoter (Figure 5A). We also found that ghrelin induces the interaction of GR with NCoR and HDAC3 corepressor in HaCaT keratinocytes treated with TNFα or flagellin (Figure 5B). Chromatin immunoprecipitation (ChIP) assay consistently demonstrated that ghrelin induces the recruitment of GR, NCoR, and HDAC3 to the nGRE site on the TSLP gene promoter (Figure 5C). However, GHRP6 or RU-486 treatment reduced the occupancy of GR, NCoR, and HDAC3 at the nGRE site on the TSLP gene promoter induced by ghrelin (Figure 5D,E).

### 2.6. Ghrelin Induces PKCδ and p300-Mediated Reduction of NF-κB p65 Acetylation

**Figure 6:**
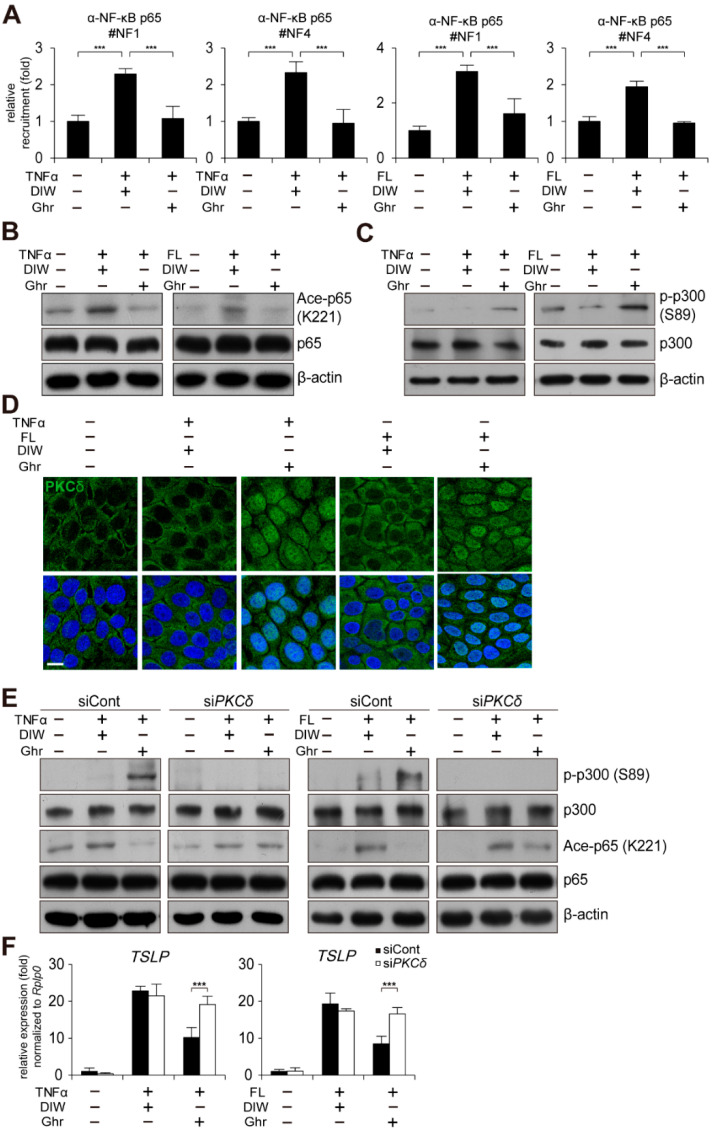
Ghrelin induces PKCδ and p300-mediated reduction of NF-κB p65 acetylation. (**A**) Ghrelin abolishes the recruitment of NF-κB p65 to the NF-κB binding sites on the TSLP gene promoter in HaCaT keratinocytes treated with TNFα or bacterial flagellin (FL). ChIP assay was performed using anti-NF-κB p65 antibody (*n* = 3). Real-time PCR was performed to quantify the occupancy of each protein at the gene promoter encompassing the NF-κB binding sites (#1 and 4). IgG was used for negative control. DIW was used as solvent for TNFα, flagellin, and ghrelin. (**B**) Ghrelin decreases acetylation of NF-κB p65 (K221) in HaCaT keratinocytes treated with TNFα or bacterial flagellin. HaCaT keratinocyte lysates were immunoblotted with anti-NF-κB p65, anti-acetyl NF-κB p65 (K221), and anti-β-actin antibodies (*n* = 3). β-actin was used as a control. Representative images are shown. (**C**) Ghrelin increases phosphorylation of p300 (S89) in HaCaT keratinocytes treated with TNFα or bacterial flagellin. HaCaT keratinocyte lysates were immunoblotted with anti-p300, anti-phospho p300 (S89), and anti-β-actin antibodies (*n* = 3). Representative images are shown. (**D**) Ghrelin induces nuclear translocation of PKCδ in HaCaT keratinocytes treated with TNFα or bacterial flagellin. HaCaT keratinocytes were immunostained with anti-PKCδ antibody (*n* = 3) (top panel). DAPI was used for nuclei staining (low panel). Scale bar, 10 μm. Representative images are shown. (**E**) Depletion of PKCδ by siRNA abolishes ghrelin-induced increase of p300 (S89) phosphorylation and decrease of NF-κB p65 (K221) acetylation in HaCaT keratinocytes treated with TNFα or bacterial flagellin. HaCaT keratinocyte lysates were immunoblotted with anti-p300, anti-phospho p300 (S89), anti-NF-κB p65, anti-acetyl NF-κB p65 (K221), and anti-β-actin antibodies (*n* = 3). Representative images are shown. (**F**) Depletion of PKCδ by siRNA abolishes ghrelin-induced suppression of TSLP gene activation in HaCaT keratinocytes treated with TNFα or bacterial flagellin. Transcripts of TSLP and RPLP0 were quantified using real-time PCR (*n* = 3). All data represent mean ± S.E.M. Significance value was *** *p* ≤ 0.005.

NF-κB is a critical transcription factor for TSLP gene activation in keratinocytes [56,57]. Thus, we examined by chromatin immunoprecipitation (ChIP) assay whether ghrelin regulates the occupancy of NF-κB p65 at the TSLP gene promoter. Ghrelin reduced the binding of NF-κB p65 to the TSLP gene promoter in HaCaT keratinocytes treated with TNFα or flagellin (Figure 6A). However, the expression level and nuclear localization of NF-κB p65 were not changed in HaCaT keratinocytes treated with TNFα or flagellin (Figure 6B and Supplementary Figure S6). We also examined acetylation of NF-κB p65 at lysine 221 (K221), which affects DNA binding activity of NF-κB p65 [58,59]. Ghrelin reduced acetylation level of NF-κB p65 (K221) in HaCaT keratinocytes treated with TNFα or flagellin (Figure 6B).

Acetylation of NF-κB p65 (K221) is catalyzed by p300 coactivator and increased phosphorylation of p300 at serine 89 (S89) inactivates acetyltransferase activity of p300 [58,60,61,62,63]. Thus, we examined phosphorylation level of p300 (S89) by Western blot analysis. Ghrelin induced increased phosphorylation of p300 (S89) (Figure 6C), suggesting that ghrelin may decrease acetylation level of NF-κB p65 (K221) and DNA binding activity of NF-κB p65 to the TSLP gene promoter through decreased acetyltransferase activity of p300.

We also examined PKCδ as a putative kinase for p300 (S89) [61,62,63,64,65,66]. We found that ghrelin induces nuclear localization of PKCδ in HaCaT keratinocytes treated with TNFα or flagellin (Figure 6D). In addition, depletion of PKCδ suppressed ghrelin-induced phosphorylation of p300 (S89) and deacetylation of NF-κB p65 (K221) (Figure 6E and Supplementary Figure S4C). Consistently, depletion of PKCδ abolished ghrelin-induced suppression of TSLP gene activation in HaCaT keratinocytes treated with TNFα or flagellin (Figure 6F).

## 3. Discussion

Ghrelin is a peptide hormone secreted from enteroendocrine cells of the gastrointestinal tract that has diverse biological functions, including appetite and obesity regulation, inhibition of insulin secretion, and stimulation of growth hormone release [67,68,69,70,71]. Ghrelin also regulates inflammation in tissue damage of the central nervous system, cardiovascular system, and gastrointestinal system [72,73,74,75,76,77,78]. In skin, it has been reported that ghrelin is expressed in several types of cells, including epidermal cells [36,79]. Qu et al., further showed decreased expression of ghrelin in TNFα-treated skin cells and inflamed mouse skin of contact dermatitis and psoriasiform [36]. They also demonstrated that intraperitoneal injection of ghrelin has therapeutic effects on both contact dermatitis and psoriasis through up-regulation of ghrelin levels in skin and attenuation of NF-κB signaling, which leads to reduced secretion of pro-inflammatory cytokines and skin inflammation [36]. Ghrelin also inhibits infiltration of inflammatory cells in skin. For example, intraperitoneal injection of ghrelin decreased the infiltration of inflammatory cells into the dermal layer in bleomycin-induced mouse scleroderma [80]. In skin burn injury, subcutaneous injection of ghrelin reduced infiltration of inflammatory cells, including leukocytes [81].

In this study, we demonstrated that topically applied ghrelin suppresses TSLP gene activation through GHSR1a in AD-like skin. GHSR, a member of the G protein-coupled receptor family, is a receptor for ghrelin [27,82]. Although two variants of GHSR (GHSR1a and 1b) are expressed in diverse tissues, GHSR1a acts as a functional receptor [45,46]. GHSR1a is highly expressed in the hypothalamus and pituitary, but it is weakly expressed in the pancreas, spleen, kidney, and adrenal gland [83,84,85]. Although it has been previously reported that GHSR1a is not detected in mouse and human skin by quantitative RT-PCR [84,85], immunohistochemical examination demonstrated that GHSR1a was present in skin epidermis and annexes surrounding neurofibromas [86]. In skin wounds, granulation tissues are positive for GHSR1a in the dermis [34]. In addition, epidermal stem cells from mouse skin express GHSR1a at the mRNA and protein level [79]. Our immunohistochemical and Western blot analysis demonstrated the presence of GHSR1a in mouse skin epidermis. We also found that HaCaT keratinocytes, a spontaneously transformed immortal keratinocyte cell line from adult human skin, express GHSR1a at the mRNA and protein level as well as GHSR1a-dependent calcium mobilization. Consistent with our results, functional GHSR1a expression has been reported in HaCaT keratinocytes [87].

We demonstrated that topically applied ghrelin-induced GHSR1a activation results in GR activation. Liu et al. suggested that ghrelin induces up-regulation of GR expression by inactivation of p38 mitogen-activated protein kinase (MAPK) and Jun N-terminal kinase (JNK), resulting in activation of anti-inflammatory gene expression and inhibition of pro-inflammatory gene expression in macrophages [34]. However, we found that ghrelin induces nuclear trans-localization and phosphorylation of GR (S211), which is required for transactivation of target genes, in HaCaT cells rather than increased GR expression [88,89]. Our results further demonstrated that activated GR by ghrelin induces binding of GR with HDAC3 and NCoR corepressor to nGRE site of the TSLP gene promoter, resulting in suppression of TSLP gene activation. Similarly, glucocorticoids suppress TSLP gene activation induced by a vitamin D3 analog through binding of GR to nGRE site [53]. The exact molecular mechanism underlying ghrelin- mediated phosphorylation and nuclear localization of GR in skin is unclear, although it is known that ghrelin may stimulate the secretion of cortisol [90,91,92,93]. It is interesting to test whether ghrelin induces secretion or synthesis of cortisol in skin which is an extra-adrenal source of cortisol synthesis [94,95]. However, ghrelin-induced increased levels of cortisol may be associated with the side effects of cortisol, such as skin atrophy [96,97]. Thus, the exact molecular mechanism of ghrelin in inflammatory skin diseases should be studied.

NF-κB is a critical transcription factor for TSLP gene activation in response to various stimuli [56,57]. We showed that ghrelin decreases occupancy of NF-κB at the TSLP gene promoter without trans-localization of NF-κB p65 into the cytoplasm or degradation of NF-κB p65 in the nucleus. This is reminiscent of our previous report that activation of aryl hydrocarbon receptor (AhR) inhibits NF-κB-dependent TSLP gene activation through PKCδ/p300-mediated reduced acetylation level of NF-κB p65 [66]. Similarly, we found that ghrelin induces nuclear localization of PKCδ, a putative p300 (S89) kinase, and increased phosphorylation of p300 at serine 89 (S89). Given that p300 acetylates NF-κB p65 (K221) and phosphorylation of p300 (S89) inhibits intrinsic lysine acetyltransferase activity of p300 [62,63], phosphorylation of p300 (S89) may be associated with the decreased DNA binding activity and transcriptional activation of NF-κB p65 [58,59,98,99,100]. Consistent with our previous study [66], we confirmed the important role of PKCδ in ghrelin-mediated negative regulation of TSLP gene activation. It has also been reported that ghrelin activates PKCδ in colon epithelial cells and dopaminergic neurons [101,102]. 

Epidermal hyperplasia is a histological hallmark promoted by various growth factors and cytokines such as keratinocyte growth factor (KGF), IL-4, IL-13, IL-17A, IL-22, and IL-24 in AD [103,104]. We found that ghrelin suppresses epidermal hyperplasia probably due to inhibition of expression of IL-4, IL-13, and IL-22 genes by ghrelin in DNFB-induced AD-like mouse skin, implying anti-proliferation activity of ghrelin (Supplementary Figure S1). Infiltration of mast cells, eosinophils, and T helper cells is also a characteristic feature of AD. Mast cells are considered the key effector cells in immediate hypersensitivity, and an increased number of mast cells is found in most AD patients [105,106]. High-affinity IgE receptor-mediated activated mast cells are responsible for secretion of various pro-inflammatory mediators, sensitization to allergens, and IgE elevation [105]. IL-4 induces production of pro-inflammatory cytokines in Th2 cells and IgE in B cells [107]. IgE then promotes infiltration of mast cells resulting in infiltration of other inflammatory cells in AD [105,106]. We further demonstrated that ghrelin suppresses IL-4 gene activation that may cause decreased infiltration of mast cells in DNFB-induced AD-like mouse skin (Supplementary Figure S1). However, it should be studied whether ghrelin suppresses IL-4, IL-13, and IL-22 gene activation, such as the TSLP gene. 

In conclusion, we identified the molecular mechanism underlying ghrelin-induced suppression of TSLP gene activation and alleviation of AD-like phenotypes. Specifically, ghrelin induces GR activation. In turn, activated GR binds to nGRE site in the TSLP gene promoter, repressing its expression. Ghrelin also induces nuclear localization of PKCδ and increases phosphorylation and inactivation of p300. This further leads to decreased acetylation and DNA binding activity of NF-κB p65 to the TSLP gene promoter (Figure 6). Collectively, our results suggest that ghrelin may alleviate inflammatory skin diseases such as AD through suppression of TSLP gene activation in skin keratinocytes.

## 4. Materials and Methods

### 4.1. Cell Culture

Human HaCaT keratinocytes were maintained in DMEM supplemented with 10% fetal bovine serum and antibiotics. To induce TSLP gene expression, cells were treated with 50 ng/mL TNFα (Bio Basic, Markham ON, Canada) or 10 ng/mL bacterial flagellin (Enzo Life Sciences, New York, NY, USA) for 1 h. Subsequently, cells were treated with 200 nM acyl-ghrelin (Peptides International, Louisville, KY, USA), 200 nM dexamethasone (Sigma-Aldrich, St. Louis, MO, USA), 1 µM [D-Lys3]-GHRP6 (Sigma-Aldrich, St. Louis, MO, USA), or 1 µM RU-486 (Sigma-Aldrich, St. Louis, MO, USA) for 1 h. TNFα, bacterial flagellin, ghrelin, and GHRP6 were dissolved in DIW. Dexamethasone and RU-486 were dissolved in ethanol.

### 4.2. Animal Model of AD

Adult female BALB/c mice (6 weeks old) (DBL, Chungcheongbuk-do, Korea) were held in a temperature-controlled room (22 °C) at 55% humidity. The committee for experimental animal research at Sogang University approved the animal experiments [IACUCSGU2017-2 (1 June 2017) and IACUCSGU2019-15 (11 September 2019)]. Dermatitis was induced by DNFB in mice as described previously [108]. The sensitization was performed once by topical application of 100 µL of 0.15% DNFB dissolved in acetone to the shaved abdominal skins of mice. A week later, the shaved dorsal skin of mice was topically applied with 100 µL of 0.15% DNFB every 3 days for 12 days. The same mice were also topically applied with 100 µL DIW, 100 µL of 100 µM acyl-ghrelin, or 1 mM [D-Lys3]-GHRP6 daily for 12 days. In the negative control group, 100 µL of acetone or DIW was topically applied to the shaved dorsal skin. As a positive control, DNFB-treated mice were topically applied with 100 µL of 200 µM dexamethasone. After mice were anesthetized with 2% isoflurane, skin lesions were photographed and harvested. Ghrelin and GHRP6 were dissolved in DIW. Dexamethasone was dissolved in ethanol.

### 4.3. Scoring AD-like Phenotypes

The score of the AD-like phenotype was calculated according to the criteria as described previously [109] with slight modifications. The severity score (0 to 12) was defined as the sum of individual scores graded as 0 (none), 1 (mild), 2 (moderate), and 3 (severe) for each of the four symptoms: (i) erythema/hemorrhage, (ii) edema, (iii) excoriation/erosion, and (iv) scaling/dryness. The maximum score was 12.

### 4.4. Calcium Mobilization Assay

Intracellular calcium levels were determined with the FLUOFORTE calcium assay kit (Enzo Life Sciences, New York, NY, USA) according to the manufacturer’s protocol.

### 4.5. Quantitative PCR

Total RNA was extracted from HaCaT keratinocytes or mouse skin using Tri-RNA Reagent (Favorgen, Ping-Tung, Taiwan). First-strand cDNA synthesis was performed with PrimeScript RT master mix (Takara, Shiga, Japan). The resulting cDNAs were subjected to real-time PCR using qPCR 2x Premix SYBR (Enzynomics, Daejeon, Korea) with a Stratagene Mx3000p qPCR machine (Agilent Technologies, CA, USA). PCR conditions used to amplify all genes were 10 min at 95 °C and 40 cycles of 95 °C for 15 s and 64 °C for 40 s. Expression data were calculated from the cycle threshold (Ct) value using the ΔCt method for quantification. RPLP0 was used for normalization. Oligonucleotides are listed in Supplementary Table S1.

### 4.6. Immunocytochemistry, Immunohistochemistry, and Histology

HaCaT keratinocytes were fixed for 10 min with 4% paraformaldehyde in PBS and permeabilized with PBST solution (0.5% Triton X-100 in PBS) for 30 min. After blocking of cells with 5% BSA in PBST solution for 1 h, cells were incubated with anti-GR (3660S, Cell Signaling, Danvers, MA, USA), anti-GHSR1a (720078, Invitrogen, Waltham, MA, USA), anti-PKCδ (SC-213, Santa Cruz Biotechnology, Dallas, TX, USA), anti-p300 (SC-584, Santa Cruz Biotechnology, Dallas, TX, USA), and anti-NF-κB p65 (SC-372, Santa Cruz Biotechnology, Dallas, TX, USA) antibodies overnight at 4 °C. Secondary antibodies conjugated to FITC (Sigma-Aldrich, Dallas, TX, USA) were used. Dorsal skin tissues were fixed with 4% paraformaldehyde in PBS overnight at 4 °C. Tissues were dehydrated, embedded in paraffin, and sectioned at 5 μm. The tissue sections were deparaffinized by 2 changes of xylene for 10 min each. Rehydration of sections was performed with 100%, 95%, 70%, and 50% ethanol in 5 min cycles. For immunohistochemistry, the tissue sections were incubated overnight at 4 °C with anti-GHSR1a (ab85104, Abcam, Cambridge, UK) antibody. DAB staining was performed using Ultravision Quanto Detection System HRP DAB (Thermo Fisher Scientific, Waltham, MA, USA) according to the manufacturer’s protocol. For histology, the sections were stained with hematoxylin (DAKO, Glostrup, Denmark), eosin (Merk Millipore, Darmstadt, Germany), or toluidine blue (Sigma-Aldrich, St. Louis, MO, USA).

### 4.7. Isolation of Epidermis and Dermis from Mouse Skin

Epidermis was prepared from mouse dorsal skin as described previously [110] with minor modifications. Briefly, the dorsal skin was scraped off subcutaneous tissue, including the fat, until the skin was semi-translucent. Then the skin was placed dermis side down in a Petri dish filled with 0.25% trypsin solution for 2 h at 32 °C. Next, the epidermis was scraped from the dermis. The epidermis and dermis were washed twice in ice-cold PBS and used for protein extraction.

### 4.8. Immunoprecipitation and Western Blot Analysis

For immunoprecipitation, HaCaT keratinocyte lysates were prepared as described previously [111]. After centrifugation of lysates, the supernatants were incubated overnight at 4 °C with the anti-GR antibody (3660S, Cell Signaling), followed by incubation with protein A agarose beads (Amicogen, Daejeon, Korea). Protein/antibody/protein A agarose beads were washed extensively and then dissolved in an SDS sample buffer. Normal IgG (12-370, Merk Millipore, Darmstadt, Germany) was used as a control. Western blot analysis was carried out using anti-GR (3660S, Cell Signaling, Danvers, MA, USA), anti-NCoR (17-10260, Merk Millipore, Darmstadt, Germany), anti-HDAC3 (ab137704, Abcam, Cambridge, UK), anti-phospho GR (S211) (4161S, Cell Signaling, Danvers, MA, USA), anti-GHSR1a (720078, Invitrogen, Waltham, MA, USA), anti-loricrin (ab24722, Abcam, Cambridge, UK), anti-collagen VI (ab6588, Abcam, Cambridge, UK), anti-p300 (SC-584, Santa Cruz Biotechnology, Dallas, TX, USA), anti-phospho p300 (S89) (SC-130210, Santa Cruz Biotechnology, Dallas, TX, USA), anti-NF-κB p65 (SC-372, Santa Cruz Biotechnology, Dallas, TX, USA), anti-acetyl NF-κB p65 (K221) (HW149, Signalway Antibody, Maryland, USA), anti-PKCδ (SC-213, Santa Cruz Biotechnology, Dallas, TX, USA), and anti-β actin (3G4-F9; AbFrontier, Seoul, Korea) antibodies.

### 4.9. Promoter Reporter Assay

Human TSLP gene promoter was amplified using primers 5′-CCCGGTACCGGGAAACTCCATTATTACACCCTT-3′ and 5′-CCGGCTAGCACACTAAACTCTTCCCA CCACGAG-3′. PCR product was cloned into pGL3 vector (Promega, Madison, WI, USA). The negative glucocorticoid response element (nGRE) site of the TSLP gene was mutated (CCTCCGGGAGA→GAACGGGAGA) [53]. HaCaT keratinocytes were transfected transiently with pGL3 plasmids containing human TSLP gene promoter in conjunction with a control thymidine kinase promoter-driven Renilla luciferase. HaCaT keratinocytes were harvested and luciferase activity was measured using a dual luciferase reporter assay system (Promega, Madison, WI, USA). Reporter activity is represented as fold activation relative to Renilla luciferase activity.

### 4.10. Chromatin Immunoprecipitation (ChIP)

ChIP was performed as described previously [66,112]. Anti-NF-κB p65 (SC-372, Santa Cruz Biotechnology, Dallas, TX, USA), (3660S, Cell Signaling, Danvers, MA, USA), anti-NCoR (17-10260, Merk Millipore, Darmstadt, Germany), anti-HDAC3 (SC-17795, Santa Cruz Biotechnology, Dallas, TX, USA), and normal IgG (12-370, Merk Millipore, Darmstadt, Germany) were used. Real-time PCR was performed with a Stratagene Mx3000P qPCR machine (Agilent Technologies, CA, USA). Oligonucleotides are listed in Supplementary Table S2.

### 4.11. RNA Interference

HaCaT keratinocytes were transfected with siRNA against human GHSR1a (M-005513-01-0005, Dharmacon, Lafayette, CO, USA), human GR (2908-1, Bioneer, Daejeon, Korea), human PKCδ (1121871, Bioneer, Daejeon, Korea), or control siRNA (d-001206-14-05, Dharmacon, Lafayette, CO, USA) using the X-treamGENE siRNA transfection reagent (Roche, Basel, Switzerland). The efficiency of knock-down of the specific gene was confirmed with real-time PCR and Western blot analysis.

### 4.12. Statistical Analyses

All quantitative data are presented as mean ± S.E.M. for three independent experiments. The differences between the two groups were evaluated by a paired t-test. Significance values were * *p* ≤ 0.05, ** *p* ≤ 0.01, and *** *p* ≤ 0.005.

## Data Availability

Not applicable.

## Supplementary Materials

The following supporting information can be downloaded at: https://www.mdpi.com/article/10.3390/ijms23073977/s1.
