# Supplementary material for: Ghrelin Represses Thymic Stromal Lymphopoietin Gene Expression through Activation of Glucocorticoid Receptor and Protein Kinase C Delta in Inflamed Skin Keratinocytes
Source: Int J Mol Sci. 2022 Apr 2;23(7):3977. doi: 10.3390/ijms23073977 (PMC8999772; doi:10.3390/ijms23073977)
Supplement: Supplementary file 1 [file ijms-23-03977-s001.zip › ijms-1632151-supplementary.pdf]

Supplemental Information

Supplemental Figures

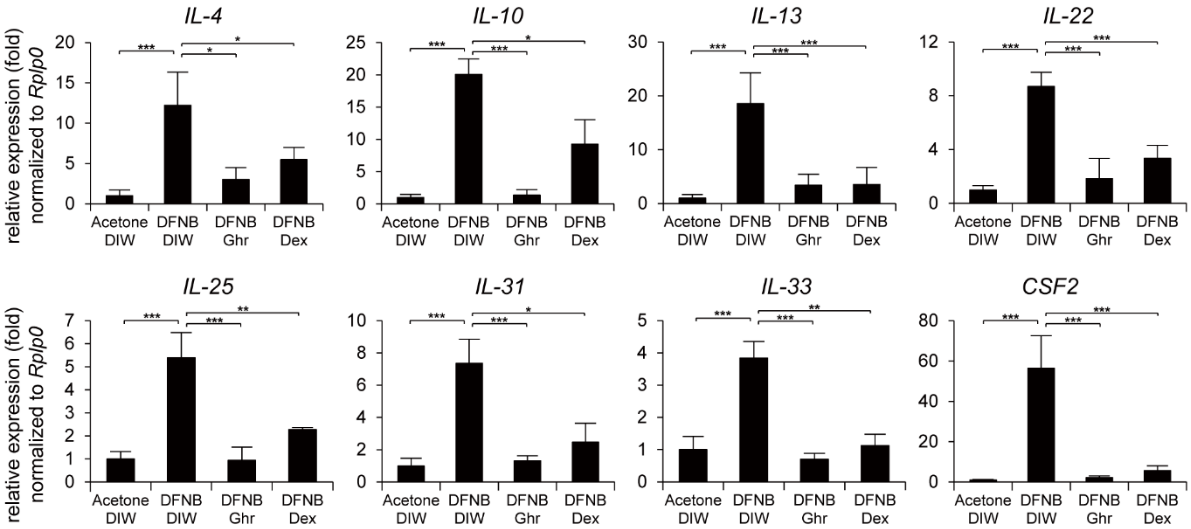

Figure S1. Ghrelin suppresses activation of cytokine gene expression in AD-like skin lesions. To induce AD-like phenotypes in mice, mice were sensitized with DNFB for 7 days and DNFB was further topically applied to the shaved dorsal skin with ghrelin for 12 days. Dexamethasone (Dex) was used as a positive control. Acetone and DIW were used as solvent for DNFB and ghrelin, respectively. Transcripts of IL-4, IL-10, IL-13, IL-22, IL-25, IL-31, IL-33, CSF2, and RPLP0 from skin were quantified using real-time PCR (n=6/group). RPLP0 was used as a control. All data represent mean ± S.E.M. Significance values were * $P \leq 0.05$, ** $P \leq 0.01$, and *** $P \leq 0.005$.

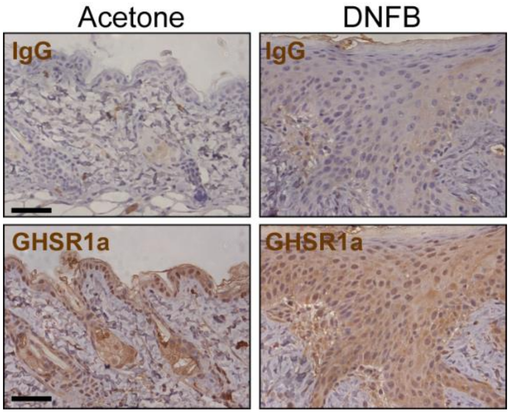

Figure S2. GHSR1a is expressed in DNFB-induced AD-like mouse skin. Skin tissue sections from control (acetone) and DNFB-treated mouse (DNFB) were immunostained with anti-GHSR1a antibody. IgG was used as a negative control. Acetone was used as a solvent for DNFB. Scale bar, 50 μm. Representative images are shown.

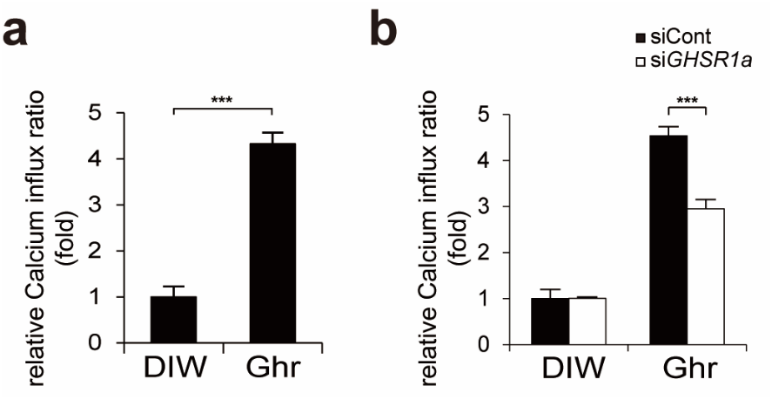

Figure S3. Ghrelin induced calcium influx is dependent on GHSR1a in HaCaT keratinocytes. (A) After HaCaT keratinocytes were treated with ghrelin for 10 min, calcium influx was measured using calcium assay kit (n=3). DIW was used as solvent for ghrelin. (B) Depletion of GSHR1a by siRNA reduces ghrelin-induced calcium influx. After HaCaT keratinocytes were transfected with control (siCont) or GHSR1a siRNA (siGHSR1a), ghrelin was treated for 10 min and calcium influx was measured (n=3). All data represent mean ± S.E.M. Significance value was *** $P \leq 0.005$.

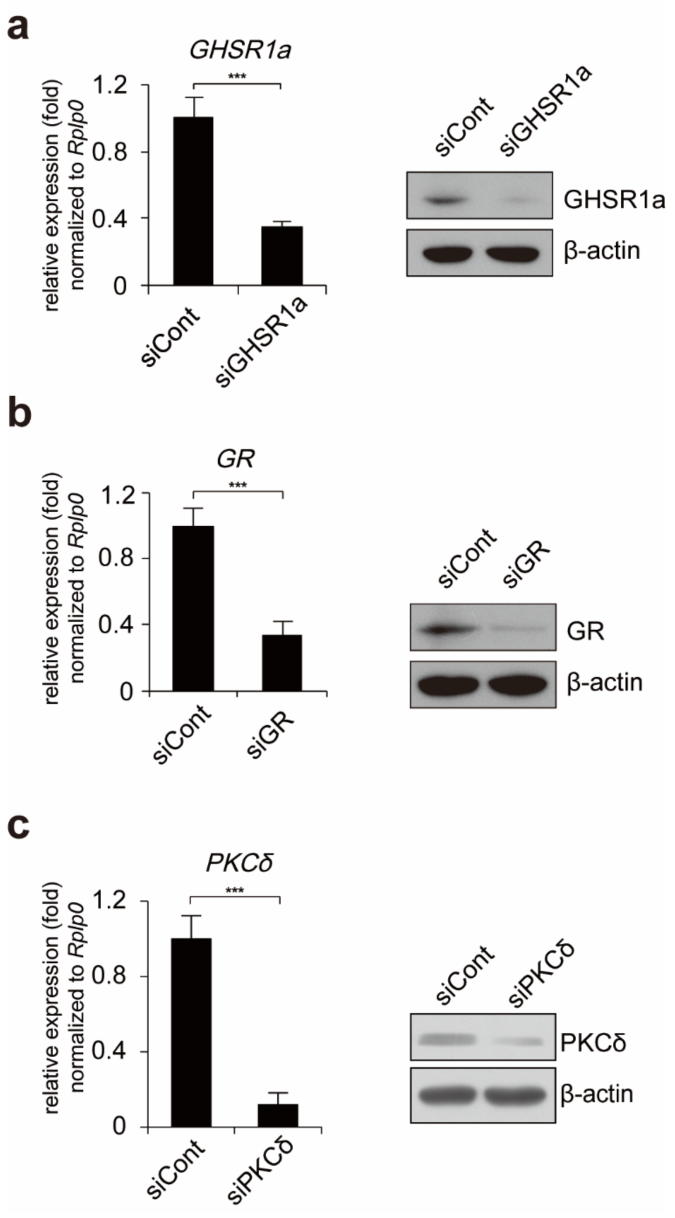

Figure S4. Efficient depletion of GHSR1a, GR, and PKCδ by RNA interference. (A~C) After HaCaT keratinocytes were transfected with control (siCont), GHSR1a siRNA (siGHSR1a), GR siRNA (siGR), and PKCδ siRNA (siPKCδ), transcripts of GHSR1a, GR, PKCδ, and RPLP0 were quantified using real-time PCR (n=3). RPLP0 was used as a control. HaCaT keratinocyte lysates were immunoblotted with anti-GHSR1a, anti-GR, anti-PKCδ, and anti-β-actin antibodies (n=3). β-actin was used as a control. Representative images are shown. All data represent mean ± S.E.M. Significance value was *** $P \leq 0.005$.

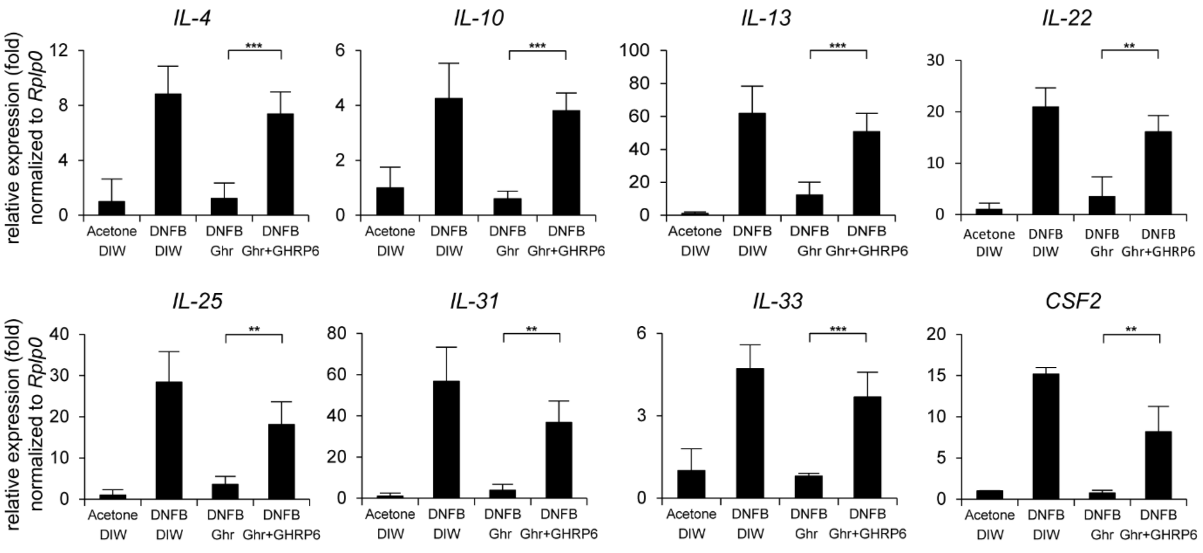

Figure S5. GHSR1a antagonist abolishes ghrelin-induced suppression of cytokine gene activation. After mice were sensitized with DNFB for 7 days, DNFB was further topically applied to the shaved dorsal skin in combination with ghrelin or GHRP6 for 12 days. Acetone was used as solvent for DNFB. DIW was used as solvent for ghrelin and GHRP6. Transcripts of IL-4, IL-10, IL-13, IL-22, IL-25, IL-31, IL-33, CSF2, and RPLP0 from skin were quantified using real-time PCR (n=6/group). RPLP0 was used as a control. All data represent mean ± S.E.M. Significance values were ** $P \leq 0.01$ and *** $P \leq 0.005$.

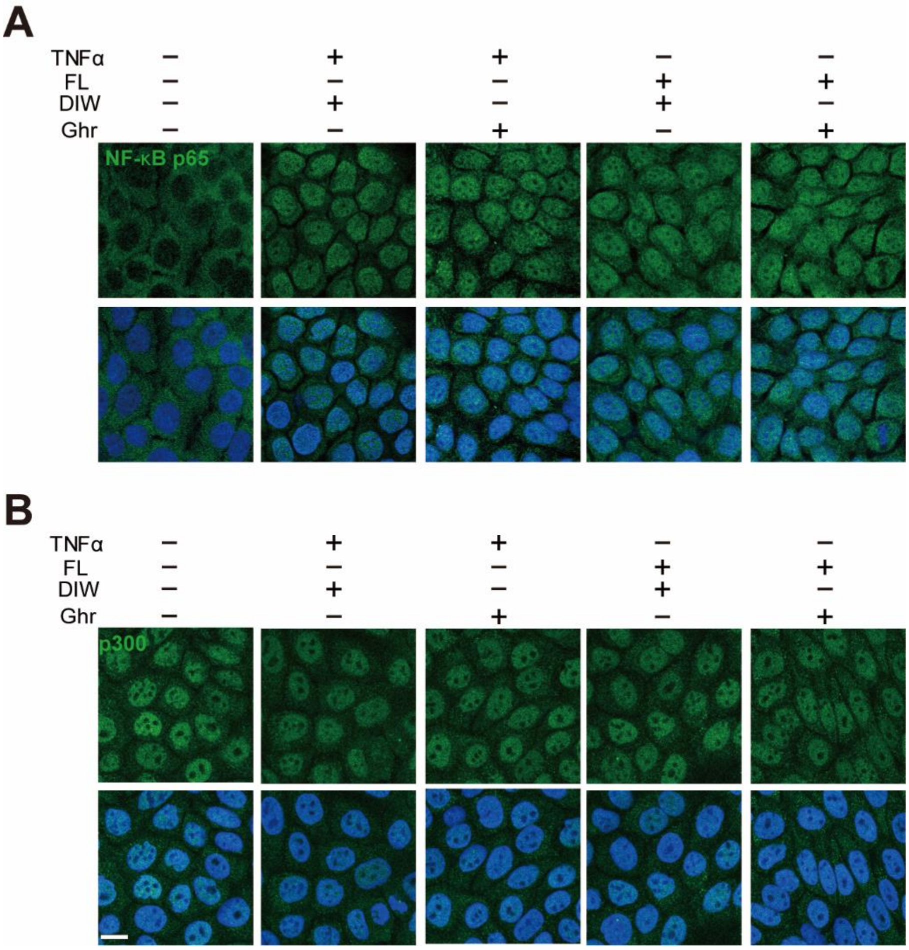

Figure S6. Ghrelin does not alter nuclear localization of NF-κB p65 and p300. (A, B) After HaCaT keratinocytes treated with TNFα or bacterial flagellin (FL), cells were treated with ghrelin. Cells were immunostained with anti-NF-κB p65 and anti-p300 antibodies (n=3). Nuclei were identified using DAPI staining. DIW was used as solvent for TNFα, flagellin, and ghrelin. Scale bar, 10 μm. Representative images are shown.

Supplemental Tables

Table S1. Oligonucleotide primers used for quantitative PCR in this study.

Gene	Forward	Reverse
**hRPLP0**	AGCCCAGAACACTGGTCTC	ACTCAGGATTTCAATGGTGCC
**hTSLP**	TATGAGTGGGACCAAAAGTACCG	GGGATTGAAGGTTAGGCTCTGG
**hGHSR1a**	TGTTTGCCTTCATCCTCTGCT	GAGGACAAAGGACACGAGGTT
**hGHSR1b**	ATGCTGTCGTGGGTGCCT	AGAGAAGGGAGAAGGCACAGG
**hGR**	ATAGCTCTGTTCCAGACTCAACT	TCCTGAAACCTGGTATTGCCT
**hPKCδ**	GTGCAGAAGAAGCCGACCAT	CCCGCATTAGCACAATCTGGA
**mRPLP**	AGATTCGGGATATGCTGTTGGC	TCGGGTCCTAGACCAGTGTTC
**mTSLP**	ACTGCAACTTCACGTCAATTACG	TTGCTCGAACTTAGCCCCTTT
**mIL-4**	GGTCTCAACCCCCAGCTAGT	GCCGATGATCTCTCTCAAGTGAT
**mIL-10**	GCTCTTACTGACTGGCATGAG	CGCAGCTCTAGGAGCATGTG
**mIL-13**	CCTGGCTCTTGCTTGCCTT	GGTCTTGTGTGATGTTGCTCA
**mIL-22**	ATGAGTTTTTCCCTTATGGGGAC	GCTGGAAGTTGGACACCTCAA
**mIL-25**	ACAGGGACTTGAATCGGGTC	TGGTAAAGTGGGACGGAGTTG
**mIL-31**	TCAGCAGACGAATCAATACAGC	TCGCTCAACACTTTGACTTTCT
**mIL-33**	GCTGCAGAAGGGAGAAATCACG	GAGTTGGAATACTTCATTCTAGGTCTCA
**mCSF2**	AGGGTCTACGGGGCAATTTC	TCACAGTCCGTTTCCGGAGTT

Table S2. Oligonucleotide primers used for ChIP in this study.

Binding Sites	Forward	Reverse
**GR (hTSLP)**	CGTTTTCCAGTCTACGCTGC	TAAGGCCAGCGACACAAGAA
**NF-κB #1 (hTSLP)**	TGAGCATATGAAAACCAAGAAG	TTGAAAAATAGTTGCCAAAAGGA
**NF-κB #4 (hTSLP)**	GGGCAAAGCAAAAAGGAGGAAG	TAAACGCCTACGGGCTCTTT
